# Advances in Therapeutic Drug Monitoring in Biologic Therapies for Pediatric Inflammatory Bowel Disease

**DOI:** 10.3389/fped.2021.661536

**Published:** 2021-05-26

**Authors:** Akshay Kapoor, Eileen Crowley

**Affiliations:** Division of Pediatric Gastroenterology, Hepatology, and Nutrition, London Health Sciences Centre, Children's Hospital Western Ontario, Western University, London, ON, Canada

**Keywords:** pediatric, inflammatory bowel disease, therapeutic drug monitoring, biologics, precision medicine

## Abstract

In the current era of treat-to-target strategies, therapeutic drug monitoring (TDM) has emerged as a potential tool in optimizing the efficacy of biologics for children diagnosed with inflammatory bowel disease (IBD). The incorporation of TDM into treatment algorithms, however, has proven to be complex. “Proactive” TDM is emerging as a therapeutic strategy due to a recently published pediatric RCT showing a clear benefit of “proactive” TDM in anti-TNF therapy. However, target therapeutic values for different biologics for different disease states [ulcerative colitis (UC) vs. Crohn's disease (CD)] and different periods of disease activity (induction vs. remission) require further definition. This is especially true in pediatrics where the therapeutic armamentarium is limited, and fixed weight-based dosing may predispose to increased clearance leading to decreased drug exposure and subsequent loss of response (pharmacokinetic and/or immunogenic). Model-based dosing for biologics offers an exciting insight into dose individualization thereby minimizing the chances of losing response. Similarly, point-of-care testing promises real-time assessment of drug levels and individualized decision-making. In the current clinical realm, TDM is being used to prolong drug durability and efficacy and prevent loss of response. Ongoing innovations may transform it into a personalized tool to achieve optimal therapeutic endpoints.

## Introduction

Biologic agents have revolutionized the treatment paradigm of pediatric inflammatory bowel disease (pIBD). Initially utilized as a second-line therapy in case of treatment failure with conventional medication (step-up approach), they are now considered as a primary induction option for children with active perianal fistulizing disease, in combination with targeted surgical intervention, as well as in children at risk of poor outcomes (top-down approach) (1, 2).

Infliximab (IFX) was the first licensed anti-tumor necrosis factor (TNF) approved for pediatric use in 2006 for treating Crohn's disease (CD) (3). It was approved for use in the pediatric population for ulcerative colitis in 2010 (4). Adalimumab, which is a fully humanized monoclonal antibody, was approved for pediatric CD in 2012 (5) and has been recently approved by the US FDA for moderate to severe pediatric UC based on the ENVISION I phase 3 study (6). With the advent of gut selective anti-integrin molecules like vedolizumab, which offers promising clinical response in colonic IBD with negligible side effects, the choice of biologic agents is increasing (7). Recently, ustekinumab, which is a biologic targeting the IL-12/23 pathway, has been approved for use in adult IBD (CD and UC) (8, 9) and is being used off label on a compassionate basis in pediatric IBD centers (10).

**Figure 1:**
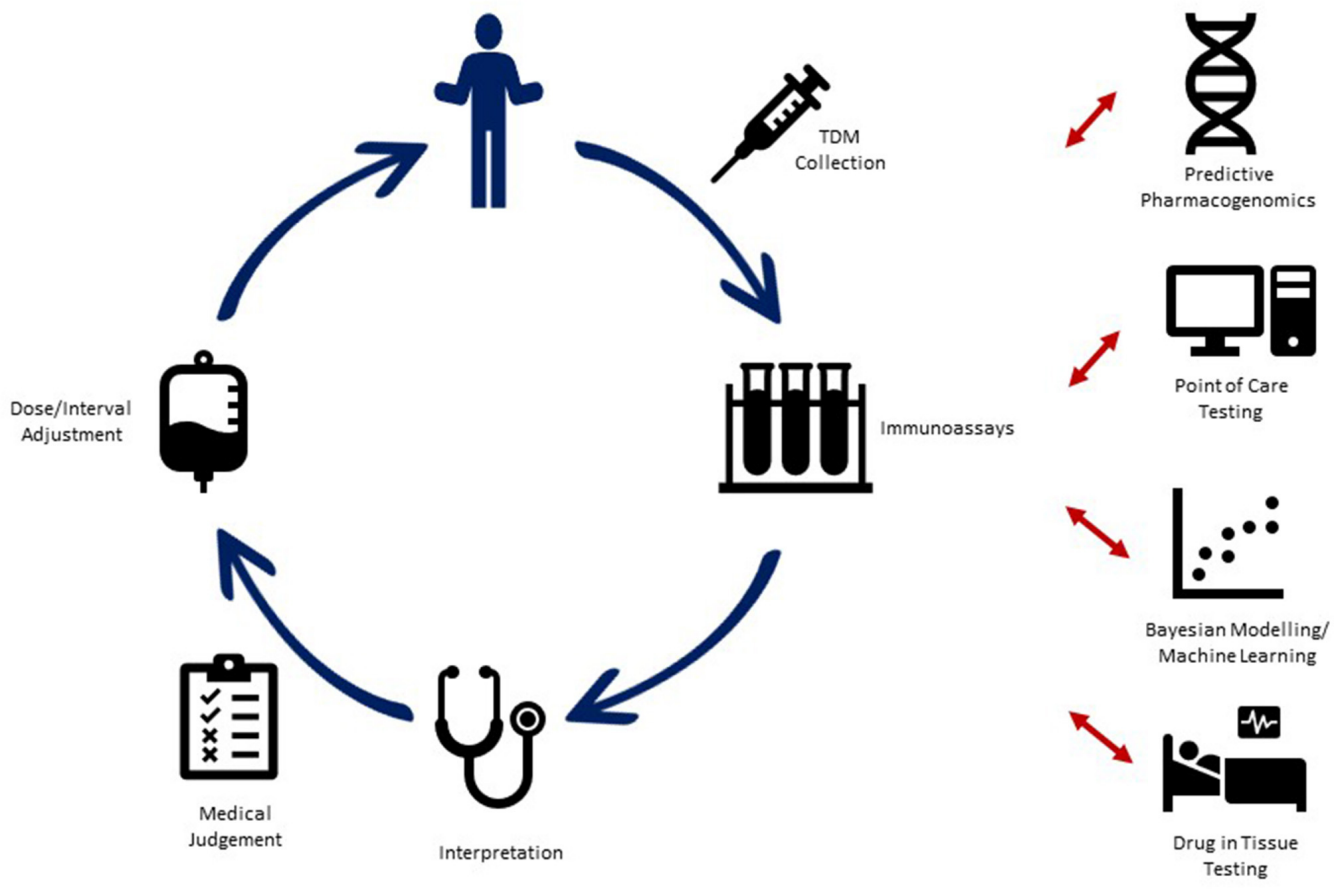
Current utilization of therapeutic drug monitoring (TDM) in pediatric IBD, along with proposed advances for novel TDM strategies into the future.

Therapeutic drug monitoring (TDM) which involves measuring drug concentration and antibody levels to optimize biologic exposure, thereby increasing efficacy and decreasing possible toxicity, is an essential tool in the arsenal to treat pIBD (Figure 1). Despite its widespread use in clinical practice, there are subtle issues which need to be addressed before uniform guidelines can be formulated. Questions around timing of TDM (proactive vs. reactive), frequency (during induction, maintenance, or both), and drug thresholds (underdosage vs. futility level) need to be aligned with the current treat-to-target paradigms (clinical, endoscopic, and histologic remission).

## Methodology

A review of the medical literature incorporating keywords was performed on databases (to include PubMed, Medline, Embase, Scopus, Web of Sciences). Attention was given to previous reviews and seminal articles and an attempt was made to include recent advances and developments in the field. All relevant articles up to December 2020 were included.

### Reactive vs. Proactive TDM

**Figure 2:**
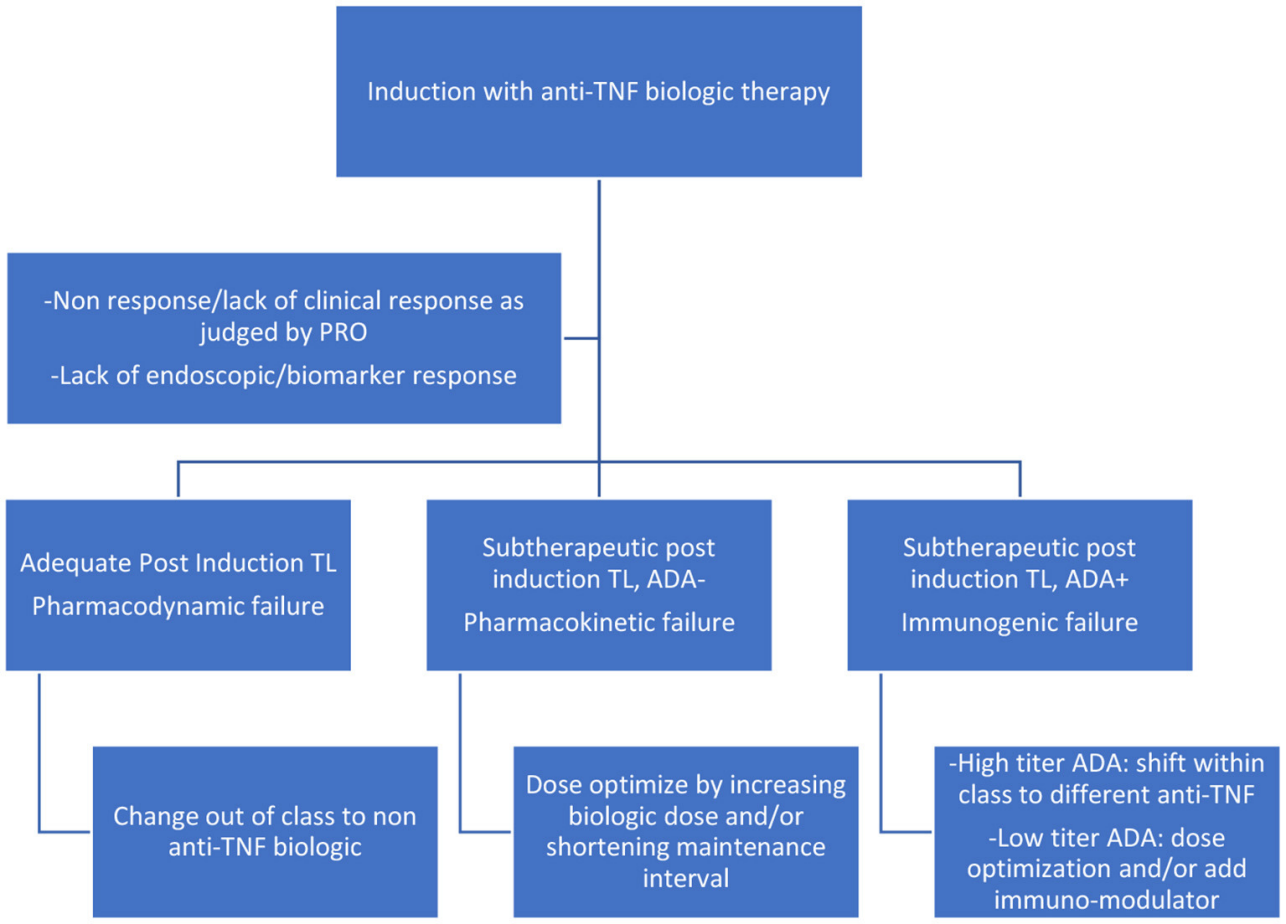
Reactive drug monitoring scenarios during induction with anti-TNF agents.

**Figure 3:**
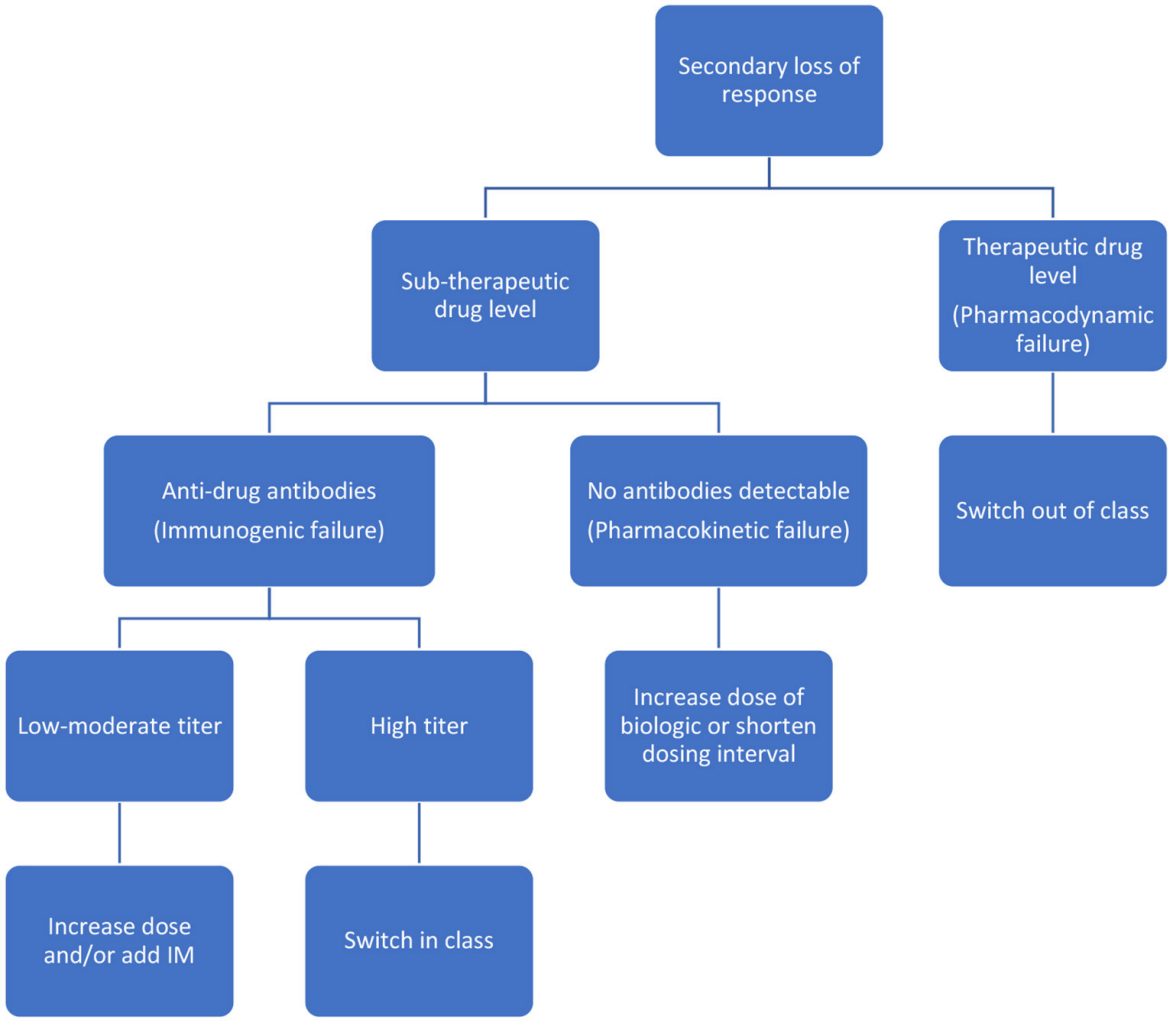
Reactive drug monitoring scenarios and management strategies during maintenance.

Reactive testing is performed in the setting of active disease/flare/intolerance to the drug. It helps in delineating the possible cause of loss of response (LOR) and in possibly formulating a strategy to counteract it (Figures 2, 3). Intuitively, higher trough levels (TL) of the drug equate to higher exposure and should theoretically result in better clinical outcomes. This has been shown with different anti-TNFs in seminal adult studies (11–13). However, most of these studies were retrospective *post hoc* analyses. Recently published prospective studies seem to have confirmed this trend (14, 15). Similarly, pediatric retrospective studies performed during the maintenance phase demonstrate an inverse association between trough level and antibody formation (16–18). Prospective pediatric studies have looked at infliximab (IFX) levels in the postinduction phase (Table 1). Adedokun et al. (19) in a phase 3 RCT concluded that higher IFX TL at week 8 correlated with better clinical and histological outcomes in children with moderate to severe UC. Singh et al. (20) highlighted the importance of a week 14 TL as an indicator for clinical and endoscopic remission at week 54. TLs performed during the induction phase have the added benefit of differentiating between a mechanistic failure (primary non-response to anti-TNF) vs. a pharmacokinetic failure due to inadequate dosing and/or increased clearance (21).

**Table 1:** Summary of pediatric studies utilizing TDM for biologic efficacy.

**Study**	**Type**	**Year**	**Drug**	**Age (years) Median (IQR)**	***N***	**Type of IBD**	**Outcomes measured**	**Results**	**ADA**	**Assay**
Adedokun et al. (19)	Prospective randomized open label	2013	IFX	14.5 (11.5–16)	60	UC	IFX TL in children through induction and maintenance	PUCAI, Mayo score IFXw8: >41.1 associated with CR and MH ~93%	4/52 (8%)	ELISA
Singh et al. (20)	Prospective observational cohort	2014	IFX	11.4 (6.6–18.4)	46	CD/UC	Postinduction TL, clinical and endoscopic outcomes	W14: 5.1 (3.1–7.5) W54: 5.2 (3.7–9.1) CR: 4.7 μg/ml vs. NR 2.6 μg/ml (*p* = 0.03)	W14: 10% W54: 26%	ELISA HMSA
Hoekman et al. (77)	Prospective cohort	2015	IFX	15 (12.9–16.3)	39	CD/UC	Maintenance TL, clinical, and biomarker remission	CR (median): 3.5 μg/ml vs. NR 2.3 μg/ml (*p* = 0.2) TL ~ CRP (*p* < 0.01) TL ~ fCal (*p* < 0.01)	4/32 (12%)	ELISA
Zitomersky et al. (78)	Cross-sectional	2015	IFX	17.3 ± 4.3	134	CD/UC	Impact of ADAs on trough levels and clinical parameters	ADA <5 U/ml: 80% ADA >5: 20% ADA >10: 13% ADA >12: 10% IFX TL + ADA >5: 1 μg/ml (1–9.3) IFX TL+ ADA <5: 12.2 μg/ml (7.6–25)	27/134 (20%)	HMSA
Sharma et al. (79)	*Post hoc* analysis of double-blind randomized study	2015	Ada	13.6 (6–17)	189	CD/UC	Relationship of induction and maintenance TL with remission	W4: CR (14.5 μg/ml) vs. NR (13.6 μg/ml), *p* = 0.28 W26: CR (11.3 μg/ml) vs. NR (10.5 μg/ml), *p* = 0.02 W52: CR (13.3 μg/ml) vs. NR (9.85 μg/ml), *p* = 0.11	6/182 (3.3%)	ELISA
Minar et al. (80)	Retrospective cohort	2016	IFX	12 ± 4	72	CD	TL for dose intensification	Undetectable TL: 24% TL <3: 38% Dose intensification on basis of TL: 35%	14/72 (19%)	ELISA HMSA
Dubinsky et al. (81)	*Post hoc* analysis of double-blind randomized study	2016	Ada	13.6 (range 6–17)	83	CD	Clinical outcomes with dose escalation based on TLs	CR before dose increase TL 9.8 μg/ml CR after dose increase TL: 21 μg/ml Cre before dose increase TL: 9.1 μg/ml Cre after dose increase TL: 19.1 μg/ml	6/182 (3.3%)	ELISA
Stein et al. (76)	Prospective cohort	2016	IFX	14.79 (12.2–16.8)	77	CD	Durability of biologic based on W10 TL	W10 TL Off IFX at 12 months: 8.7 μg/ml On IFX at 12 months: 20.4 μg/ml	18/77 (23%)	HMSA
Choi et al. (16)	Retrospective cohort	2017	IFX	14.7 (9–18.8)	39	CD/UC	Maintenance TL and clinical remission	CR (median): 3.99 μg/ml vs. NR (median): 0.88 μg/ml, *p* = 0.02	7/39 (18%)	ELISA
Merras-Salmio et al. (17)	Retrospective cohort	2017	IFX	14.8 (12.5–16)	146	CD/UC	Maintenance TL and clinical remission	CR (median): 3.7 μg/ml vs. NR (median): 1.2 μg/ml, *p* = 0.005	52/208 (25%)	ELISA
Rolandsdotter et al. (18)	Retrospective cohort	2017	IFX	16 (7–18)	45	CD/UC	Maintenance TL, clinical, and biomarker remission	CR (median): 7.2 μg/ml vs. NR (median): 1.2 μg/ml *p* < 0.05 TL ~ CRP (*p* = 0.008) TL ~ ESR (*p* = 0.003) TL ~ albumin (*p* = 0.0005)	8/45 (18%)	ELISA
Chi et al. (82)	Prospective observational cohort study	2018	IFX	18.5 ± 4.4	223	CD/UC	Combination IM + IFX therapy and relation to TL and ADA	Combination TL: 17 ± 1.33 μg/ml vs. monotherapy TL: 13.18 ± 1.26 μg/ml (*p* < 0.01)	9.5% for combination therapy vs. 20% on monotherapy	HMSA
van Hoeve et al. (83)	Retrospective cohort study	2018	IFX	12.2 (9.5–14.4)	52	CD/UC	Maintenance TL, clinical, endoscopic, and biomarker remission	TL (remission vs. not) *p* < 0.01 for all CR: 5.4 vs. 4.2 μg/ml BR: 5.2 vs. 4.2 μg/ml CR+BR: 5.7 vs. 4.4 μg/ml ER: 6.5 vs. 3.2 μg/ml	Not reported	
Ohem et al. (84)	Prospective observational study	2018	IFX	12.6 (10.5–15.1)	65	CD	TL and biomarker remission	TL for CRP <5mg/l: >1.1 μg/ml fCal <100: 3.5 μg/ml	Low TL were associated with high ADA (OR 0.027) 95% CI 0.009–0.077	ELISA
van Hoeve et al. (85)	Retrospective cohort study	2019	IFX	11.6 (8.8–13.9)	35	CD/UC	Postinduction TLs as predictors of clinical and biological remission at W52	Postinduction TL (remission vs. not), *p* < 0.002 CR: 4.6 vs. 1.5 μg/ml BR: 4.6 vs. 2.6 μg/ml CR+BR: 6.0 vs. 2.6 μg/ml	Not reported	
Assa et al. (27)	Non-blinded randomized control trial	2019	Ada	14.3 ± 2.6	78	CD	Reactive vs. proactive drug monitoring and relation with sustained corticosteroid-free CR	CFCR (8–72 weeks) 82% in the proactive group vs. 48% in the reactive group (*p* = 0.02) CFCR+BR: 42% in the proactive group and 12% in the reactive group (*p* = 0.03)	8/78 (10.2%)	ELISA
Choi et al. (86)	Retrospective cohort study	2019	IFX	14.5	103	CD/UC	Correlation of IFX levels with hematological remission (CRP, ESR, albumin, and hematocrit)	Week 6 IFX level 9.82 μg/ml was required to maintain CRP <0.5 (AUC 0.88) Week 14 IFX level of 1.28 μg/ml was required to maintain CRP <0.5 (AUC 0.86)	Not reported	ELISA
Naviglio et al. (87)	Prospective observational study	2019	IFX	14.4 (11.6–16.2)	49	CD/UC	CR as defined by PUCAI/PCDAI IFX TL and ADA (only if TL <1.5) at weeks 6, 14, 22, and 54	CR week 14, 76.3% CR week 54, 73.9% IFX level at the end of induction week 14, >3.11 μg/ml was strongest predictor of CR at week 54	10/49 (20.4%)	ELISA
Clarkston et al. (88)	Prospective observational study	2019	IFX	14.4	72	CD	CR: wPCDAI at the fourth infusion BR: >50% reduction in fCal Maintenance IFX level >5 μg/ml	CR: 64% BR: 54% Start of maintenance >5 μg/ml: 22% Infusion 2 level >29 μg/ml and infusion 3 >18 μg/ml strongly associated with improved early outcomes	Not reported	ELISA
Gofin et al. (89)	Retrospective cohort study	2020	IFX and Ada	12.6 (10.1–14.2)	197	CD	Effect on disease outcomes with TDM vs. without Drug retention Hospitalization rate/year Treatment intensification Surgical resection	Longer retention time with TDM Lower hospitalization rate with TDM Higher drug intensification rate with TDM No difference in surgical outcomes	Not reported	ELISA
Choi et al. (90)	Prospective cohort study	2020	ADA	14.1 ± 2.0	17	CD	CR based on PCDAI at week 16 MH at week 16 HR at week 16	Ada TL was higher in those with MH (13 ± 6.5 vs. 6.2 ± 2.6 μg/ml; *p* = 0.02) Ada TL was higher in those with HR (17.9 ± 5.3 vs. 6.8 ± 2.5 μg/ml; *p* = 0.02) Optimal TL for MH at week 16, 8.76 μg/ml	0%	ELISA

*PUCAI, Pediatric Ulcerative Colitis Activity Index; IFX, infliximab; Ada, adalimumab; ADA, anti-drug antibody; TL, trough level; CR, clinical remission; NR, non-remission; Cre, clinical response; BR, biomarker remission; ER, endoscopic remission; fCal, fecal calprotectin; CFCR, corticosteroid-free clinical remission; MH, mucosal healing; HR, histologic remission*.

Proactive TDM is performed in patients with quiescent disease to decrease the risk of disease relapse, treatment failure, and drug immunogenicity. It also may help in optimizing monotherapy with the biologic agent without the need for an immunomodulator, thereby avoiding potential toxic therapy. This has been examined in adult studies carried out by Lega et al. (22) and *post hoc* analysis of the SONIC trial (23). Papamichael et al. (24) in a multicentric retrospective study showed benefit for the proactive TDM approach with regard to treatment failure, disease-related hospitalization, surgery, and infusion reactions. The results of the two randomized control trials completed in adults, comparing proactive TDM to empiric optimization, have been disappointing. The TAXIT study (25) randomized IBD (UC and CD) patients on stable maintenance therapy with IFX to receive further dosing based on proactive monitoring or clinical symptoms. Interestingly, all patients were dose optimized to a TL of 3–7 μg/ml prior to randomization. At the end of 12 months, there was no difference between the groups with regard to clinical/biochemical remission. There was also no differences noted with regard to surgery and corticosteroid-free remission between the groups. The TAILORIX trial (26) included biologic naive CD adult patients starting IFX therapy and randomized them to three arms based on clinical symptoms alone, clinical symptoms and biomarkers, and clinical symptoms, biomarker, and/or IFX trough levels. Dose escalation of IFX was based on predefined criteria in the first two groups. The primary endpoint of the study was corticosteroid-free clinical remission, fistulae, or need for surgery between weeks 22 and 54. There was no difference between the groups with regard to the primary or secondary endpoints. The PAILOT (27) study is the only pediatric RCT comparing proactive and reactive TDM using adalimumab as the biologic of choice. The proactive approach demonstrated a clear benefit with regard to corticosteroid-free clinical remission between weeks 8 and 72. A recent retrospective pediatric paper showed the benefit of a proactive anti-TNF TDM approach in improving outcomes related to steroid-free clinical remission (28).

The AGA guidelines issued in 2017 recommended the use of reactive TDM to guide treatment decisions based on a very low grade of evidence; however, it refrained from making any recommendations on proactive TDM (29). A second guideline issued as a result of a Delphi process among 25 international IBD experts recommended reactive TDM for both primary non-response (PNR) and LOR. However, it also recommended proactive TDM for patients in remission immediately post induction and those on stable maintenance, to save costs (30). Systematic reviews and meta-analysis performed to resolve this deadlock have consistently shown no clear benefit of any TDM strategy over empiric optimization. The reviews have shown a consistent cost benefit with a reactive TDM strategy vs. empiric escalation (31) and drug durability benefit with the proactive strategy (32, 33). This assumes greater importance in pediatrics as IBD specialists try to factor in insurance coverage and payor–payee concerns into pharmacotherapeutic decision-making (34).

### TDM and the Newer Biologics

The α4β7 anti-integrin vedolizumab (VDZ) has been documented to have an exposure–response relationship similar to the anti-TNFs for both UC (GEMINI-1) (35) and CD (GEMINI-2) (36). *Post hoc* analysis of the GEMINI trials completed by Rosario showed that remission rates associated with TL <17 μg/ml for UC and <16 μg/ml for CD were similar to placebo (37). These findings were subsequently confirmed in two real-world cohort studies. In a Belgian study by Dreesen et al. (38), cutoff points for trough levels were calculated using AUROC during both induction and maintenance. Similarly, Ungaro et al. (39) confirmed that higher TLs of VDZ were associated with steroid-free clinical remission.

The GEMINI trials also showed the low immunogenic potential of VDZ with persistent anti-drug antibody rates <1% (31, 32). This has been confirmed in subsequent studies wherein adding an immunomodulator to VDZ therapy neither enhanced drug levels nor regained therapeutic response (40). Studies have shown near complete saturation of the α4β7 at drug levels as low as 1 μg/ml; therefore, it is currently unclear as to how dose optimization may help in recapturing therapeutic response (41). A recent abstract presented at the Digestive Diseases Week (DDW) virtual meeting suggested that colonic tissue VDZ concentration varies inversely with the severity of inflammation. Thus, increasing the TL might enable more drug to penetrate the inflamed tissue and help with clinical remission/endoscopic healing (42). While evidence is emerging that TL for VDZ may be associated with clinical and endoscopic remission (43), there are no clinical guidelines regarding the levels that need to be targeted. The ECCO-ESGAR committee recommends TDM for VDZ whenever available (44). Studies on pediatric TDM with respect to VDZ are limited. A recent Dutch study highlighted the exposure–efficacy relationship of VDZ in a pediatric IBD population which had failed anti-TNF. The authors concluded that a lower TL of VDZ in Crohn's disease patients vs. UC/IBD-U was due to the transmural nature of the disease and may benefit from proactive TDM and subsequent higher dosing (45).

Ustekinumab (UST) is an IL-12/23 inhibitor which works by blocking the common p40 subunit. The exposure–efficacy relationship for UST dosing was proven in the pivotal studies IM-UNITI and UNIFI in CD and UC, respectively (8, 9). A real-world study by Battat et al. (46) showed that a TL >4.5 μg/ml during maintenance was associated with a biomarker and endoscopic response. Of note, the majority of patients in this study had been dose optimized to receive UST every 4 weeks, against the conventional 8-week dosing. Adedokun et al. in their study with more conventional eight-weekly maintenance dosing in adult CD patients showed a TL >0.8 μg/ml to be associated with prolonged clinical and endoscopic remission (47). The immunogenic potential of UST, similar to VDZ, is also quite low; consequently, the addition of azathioprine/methotrexate is seldom required. There is still a lot of variability in the optimization protocols for UST. The seminal extension study (IM-UNITI) (48) suggested 12-weekly maintenance dosing in biologic naive patients and 8-weekly dosing in anti-TNF therapy-experienced patients. Other ongoing trials include STARDUST (treat-to-target vs. routine case management in CD patients on UST), POWER (efficacy and safety of UST reinduction therapy in patients with moderate and severe CD), and RESCUE (loss of response to UST treated with dose escalation), which might help in guiding formulation of desired TLs for different therapeutic endpoints (49).

Although, pediatric data are scarce, Dayan et al. (10) in a real-world pediatric cohort had reported 50% steroid-free remission (for biologic exposed) and 90% steroid-free remission (biologic naive) at 1 year. There was no significant difference in the trough levels between the patients on or off steroids at 52 weeks.

## Novel TDM Concepts

### Personalized Medicine—Model-Informed Precision Dosing

Currently, most of the biologic agents are administered on the basis of fixed dosing or weight-based dosing algorithms. Utilizing a TDM-based approach to look at pharmacokinetic profiles is helpful but not perfect as several factors (weight, disease load, disease stage, drug clearance) may influence the eventual TL for an individual. Adaptive dosing dashboards based on population pharmacokinetic models as a backbone are being increasingly used. The individual patient's characteristics and drug level measurements can be added on to this base model, to predict subsequent dosing requirement and frequency. While retrospective studies (50, 51) have validated the proof of concept, the PRECISION trial by Strik et al. (52, 53) was the first to employ this approach prospectively to IFX dosing. In this study, model-based dosing was superior to conventional dosing in maintaining remission. Subsequent studies performed by Dubinsky et al. (54, 55) have employed precision dosing models (Bayesian population-based pharmacokinetic model with weight, albumin, CRP, previous drug, and antibody levels added) to aim at prespecified individual TLs. Drug dosage and interval to next infusion was thus individualized based on this adaptive Bayesian modeling.

The Bayesian modeling approach seems to be a very promising development. Precision dosing has been used previously by clinical pharmacists for antibiotic dosing especially vancomycin and piperacillin–tazobactum (56, 57). However, the modeling requirements for a chronic disease like IBD are different. The efficacy of the model depends on the number of variables incorporated, and it will require constant updating and streamlining. Also, the target TL which the model aims to achieve is a moving target which varies with the disease phenotype and the disease phase. Although, it has the potential to eventually decrease drug costs by permitting de-escalation, in the short term, it will require more manpower, training, and coordination between health care teams and the pharmaceutical industry. Finally, more RCTs are needed especially during the induction phase to prove that model-informed precision dosing will be beneficial when parameters like TL and ADA are not available.

### Point-of-Care Testing

Drug levels are currently processed in labs and are usually processed in batches. This leads to an increased turnover time and is particularly challenging in certain disease states associated with unpredictable pharmacokinetics (acute UC, fistulizing CD) or during induction of therapy, especially when suspecting non-response.

Point-of-care assays can help in decreasing the turnaround time and help in providing an accurate, clinically relevant, real-time value to aid in clinical decision-making. Point-of-care assays for detecting IFX levels, adalimumab levels, and anti-IFX antibodies are commercially available. However, they are limited by their ability to use serum instead of blood (58–60). As with other point-of-care tests, there are concerns around quality control, reliability, cost, and external validation. Some of these issues were addressed by a pilot project published as an abstract by Bossuyt et al. (61), wherein, they used an ultra-proactive point-of-care TDM approach to demonstrate applicability and effectiveness. Curci et al. (62) in a pediatric study validated two point-of-care IFX assays and compared them with previously validated ELISA assays for measuring trough levels. The group observed good intraclass as well as interclass correlation.

### Therapeutic Drug Monitoring in VEOIBD

The proportion of children aged <6 years with a new diagnosis of IBD is increasing (63). The disease in younger children is more extensive, usually colonic and requires optimized treatment regimens, including the use of biologic drugs (64). It has been shown that standard IFX regimens and trough levels may not be applicable in this age group (65, 66) and may require more frequent escalation of therapy (67). This may be in part related to the size of the antigenic sink at the start of treatment or to the increased clearance related to the low body weight. Studies have also shown how weight-based dosing systematically underdoses children with low body weight (68). A recent case series by Assa et al. (69) showed how an accelerated induction protocol (increased dosing and interval shortening) helped to recapture response in a group of children with infantile IBD after they had experienced initial non-response/secondary LOR with IFX.

However, the overall clinical management of VEOIBD using biologics may be more complex than adjusting for age or weight. A recent study by Jongsma et al. (70) showed suboptimal TL for patients <10 vs. >10 years at the beginning of maintenance therapy. The former group also had a significantly higher antibody titer contributing to immunogenicity and, consequently, a lower TL. Multivariate analysis did not reveal a direct influence of age on TL. Proactive TDM in the younger age group did not seem to affect clinical remission rates at 52 weeks when compared with children >10 years.

### Genomic Variants Influencing Trough Levels

The PANTS consortium tried to look at genomic variants which could influence antibody formation and, consequently, lead to lower TL and loss of response for IFX and ADA. The group performed a GWAS to identify genomic variants that were associated with immunogenicity. They concluded that the HLA-DQA1^*^05 allele significantly increased immunogenicity with a HR 1.9 (1.6–2.25) (71).

### Tissue Drug Levels in Pediatric IBD

The ATLAS study demonstrated that anti-TNF concentration in tissue correlates with the degree of endoscopic inflammation, except in tissue with severe inflammation in which anti-TNF levels were lower (72). Tissue drug levels have not as yet been evaluated in pediatric IBD and may offer further insight into the individual's target trough level to achieve mucosal healing as a therapeutic endpoint.

## Discussion

The treat-to-target strategies recommended by the STRIDE (73) IOIBD working group aim at composite endpoints of both clinical/patient-reported remission as well as endoscopic and/or radiological healing. TDM may be intuitively linked to this goal but cannot be included in this approach due to failure of the seminal RCT studies TAXIT and TAILORIX to meet their primary endpoint/s. The lack of well-powered, prospective RCTs means that there is a knowledge gap which needs to be addressed. Meta-analysis and systematic reviews on the role of TDM are limited by the heterogeneity in study design, timing, and use of TDM, combination of different disease phenotypes, and endpoints of the different trials. Thus, many of the recommendations that emerge regarding the use of TDM for treat-to-target are based on low-grade evidence. Encouragingly, the recent PAILOT study has been helpful in proving that proactive TDM in biologic naive children on adalimumab did better than reactive TDM in the control group with regard to corticosteroid-free remission.

Trough levels in clinical practice are intended for guidance. They can vary depending on the preselected endpoint (clinical remission vs. healing based on endoscopy and histology), disease severity, size of antigenic sink, and phase of treatment (induction vs. maintenance). Higher TLs may be aimed for in children with VEOIBD (61), those with perianal fistulae (74), severe UC (75), and during induction (20, 76). LOR scenarios are taxing for both the patient and the IBD physician. While drug levels are standardized ELISA tests that can be compared between different assays, the antidrug antibody tests are not standardized and various assays differ in cutoffs for low, intermediate, and high titer antibodies. Combining the lack of clarity on TLs and the varied recommendations on reactive and proactive TDM, this is an area of research that will require further clarification in the future. Proactive TDM is emerging as a new therapeutic strategy in pediatric patients; however, prospective interventional clinical trials looking at early induction and maintenance levels for novel biologic agents with endoscopic outcomes and/or composite surrogate outcomes are needed to advance our knowledge in the pediatric population.

Interest has been growing in personalizing treatment strategies by using predictive pharmacogenomics and machine learning to propose individualized treatment protocols. Exciting developments especially related to adaptive dosing dashboards utilizing Bayesian models herald the future of personalized medicine in IBD. If successful, they have the capacity to eliminate dosing tables and target TL values and possible inherent treatment bias of the physician. Coupled with point-of-care TDM and biomarker testing, this has the potential to revolutionize how pediatric IBD will be treated in the coming years.

## Author Contributions

AK and EC contributed to the design and implementation of this review article, to the analysis of the published studies, and to the writing of the manuscript. Both authors contributed to the article and approved the submitted version.

## Conflict of Interest

The authors declare that the research was conducted in the absence of any commercial or financial relationships that could be construed as a potential conflict of interest.
